# Successful Retrieval of an Impacted Biliary Extraction Basket Without a Salvage Device: A Novel Modified Device

**DOI:** 10.1002/ccr3.70292

**Published:** 2025-03-04

**Authors:** Peixi Liu, Qin Zhang, Yufan Ma

**Affiliations:** ^1^ Department of Endoscopy Medicine People's Hospital of Sichuan Province Chengdu Sichuan China; ^2^ Department of Gastroenterology The First People's Hospital of Liangshan Yi Autonomous Prefecture Xichang Sichuan China

**Keywords:** basket impact, choledocholithiasis, complication, ERCP

## Abstract

During an ERCP procedure for bile duct stones, the patient experienced basket impaction during the operation. We successfully removed the impacted reticular basket without complications, utilizing a temporary salvage device modified with a lithotripter, thereby avoiding other future procedures such as ESWL or surgery.

1

A 39‐year‐old female presented with epigastric pain lasting for the past 3 days. Her past medical history included a laparoscopic cholecystectomy. Physical examination revealed epigastrium tenderness and mild jaundice, while other physical examinations were normal. Computed tomography (CT) of the abdomen and MRCP indicated small stones at the lower end of the CBD with obstruction and dilatation of the upper biliary system. Liver function tests were significantly elevated, showing total bilirubin 206.9 (3.4–20.5 μmol/L), direct bilirubin 161.9 (1.7–6.8 μmol/L), indirect bilirubin 45.0 (< 14.2 μmol/L), alanine aminotransferase 879 (5–40 U/L), aspartate aminotransferase 481 (8–40 U/L). C‐reactive protein 33.18 mg/L. No abnormality was found in other laboratory tests.

She underwent an ERCP; after biliary sphincterotomy, the hexagonal reticular basket successfully captured the stone; however, it got impacted at the CBD and cannot be released due to the poorly expandable (PE) CBD, though it was not detected before the operation [Figure 1]. Without using a standard salvage device, mechanical lithotripsy was performed using the metal sheath of the lithotripter (BML‐V232QR‐30, Olympus, Φ2.9 mm). After cutting off the plastic handle of the basket and lithotripter [Figure 2], the plastic sheath of the basket was replaced with the metal sheath of the lithotripter via the basket wires under direct endoscopic visualization [Figure 3A,B]. A wrench was used to hold the metal sheath, and a vise was used to tighten the basket wires until the trapped stone within the basket was crushed [Figure 3C–E]. Finally, the basket was successfully removed without complications, avoiding additional procedures such as Extracorporeal Shock Wave Lithotripsy (ESWL) or surgery.

**FIGURE 1:**
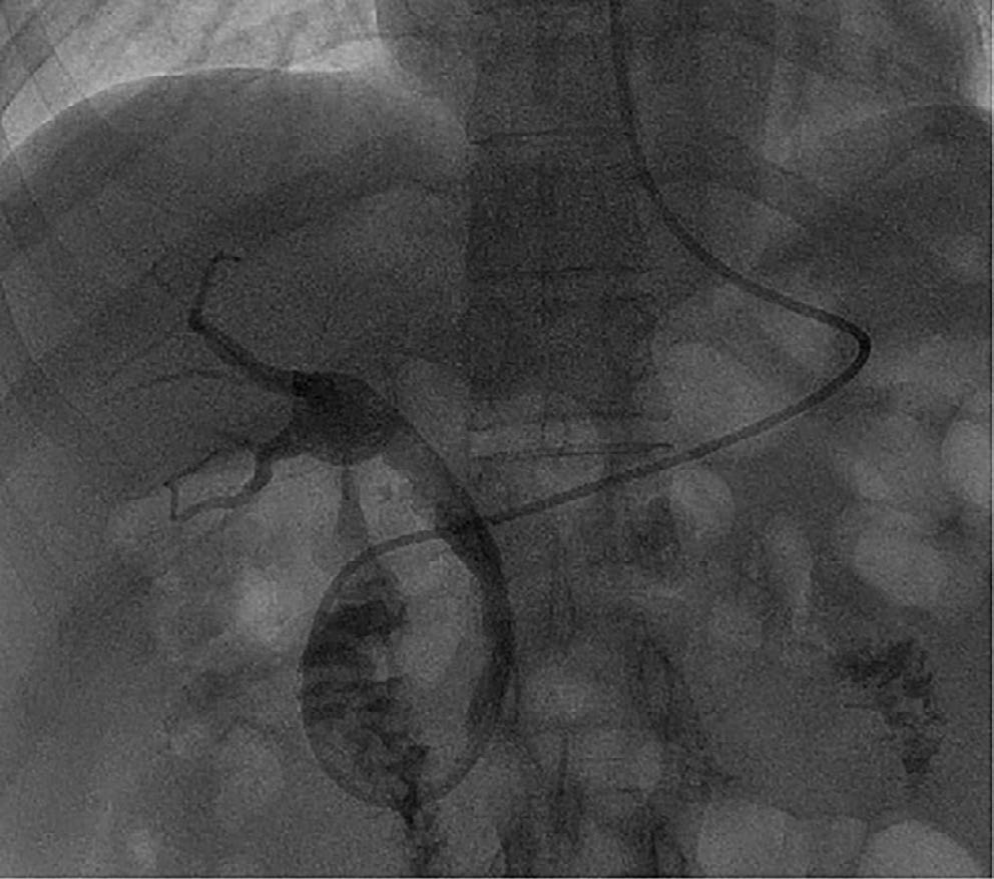
Postoperative cholangiography through nasobiliary catheter suggested PE CBD.

**FIGURE 2:**
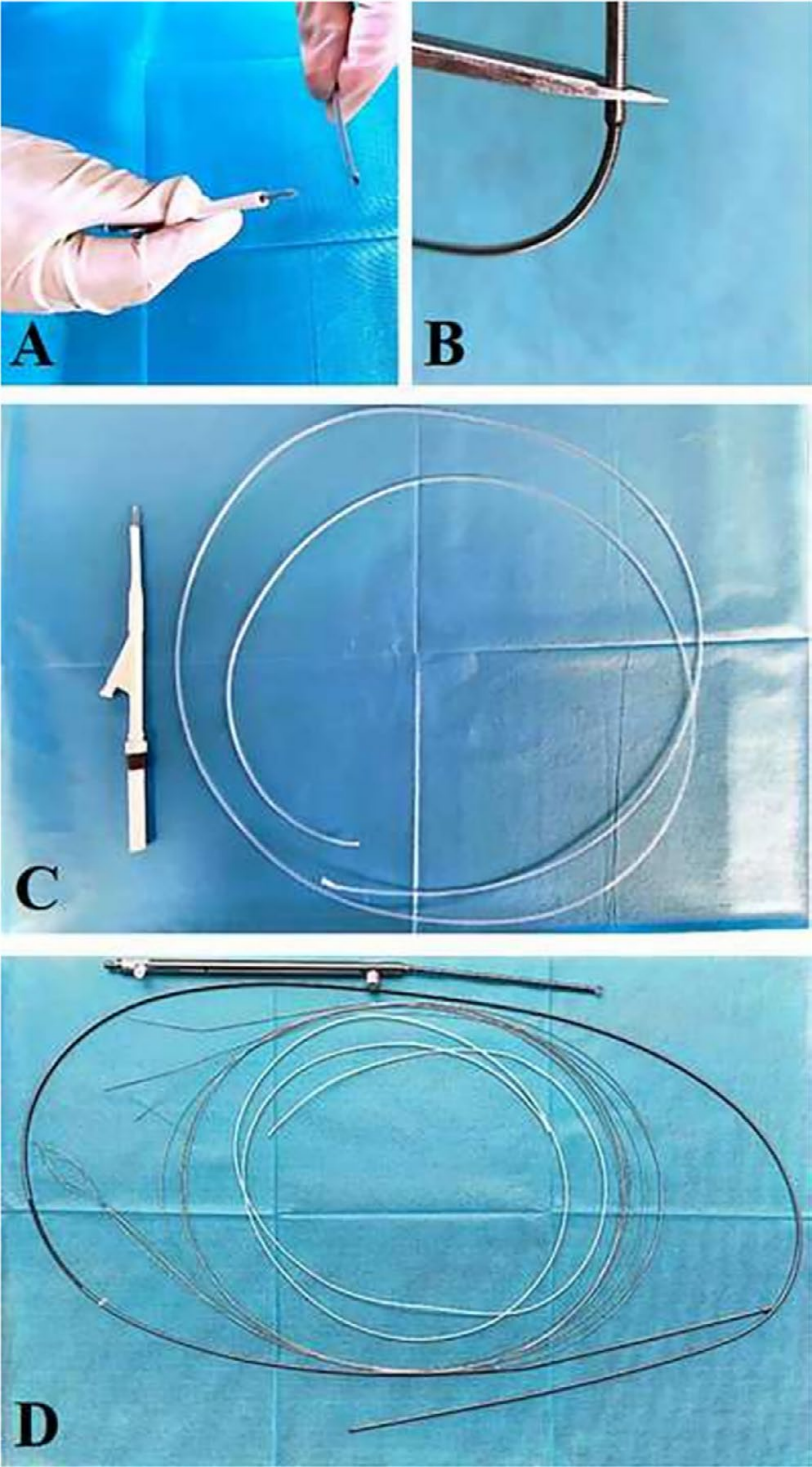
(A) Cutting off the reticular basket from the handle. (B) Cutting off the lithotripter from the handle. (C) The handle and plastic sheath of the reticular basket. (D) From top to bottom: Lithotripter handle, metal outer sheath, inner core, and plastic sheath covering the inner core.

**FIGURE 3:**
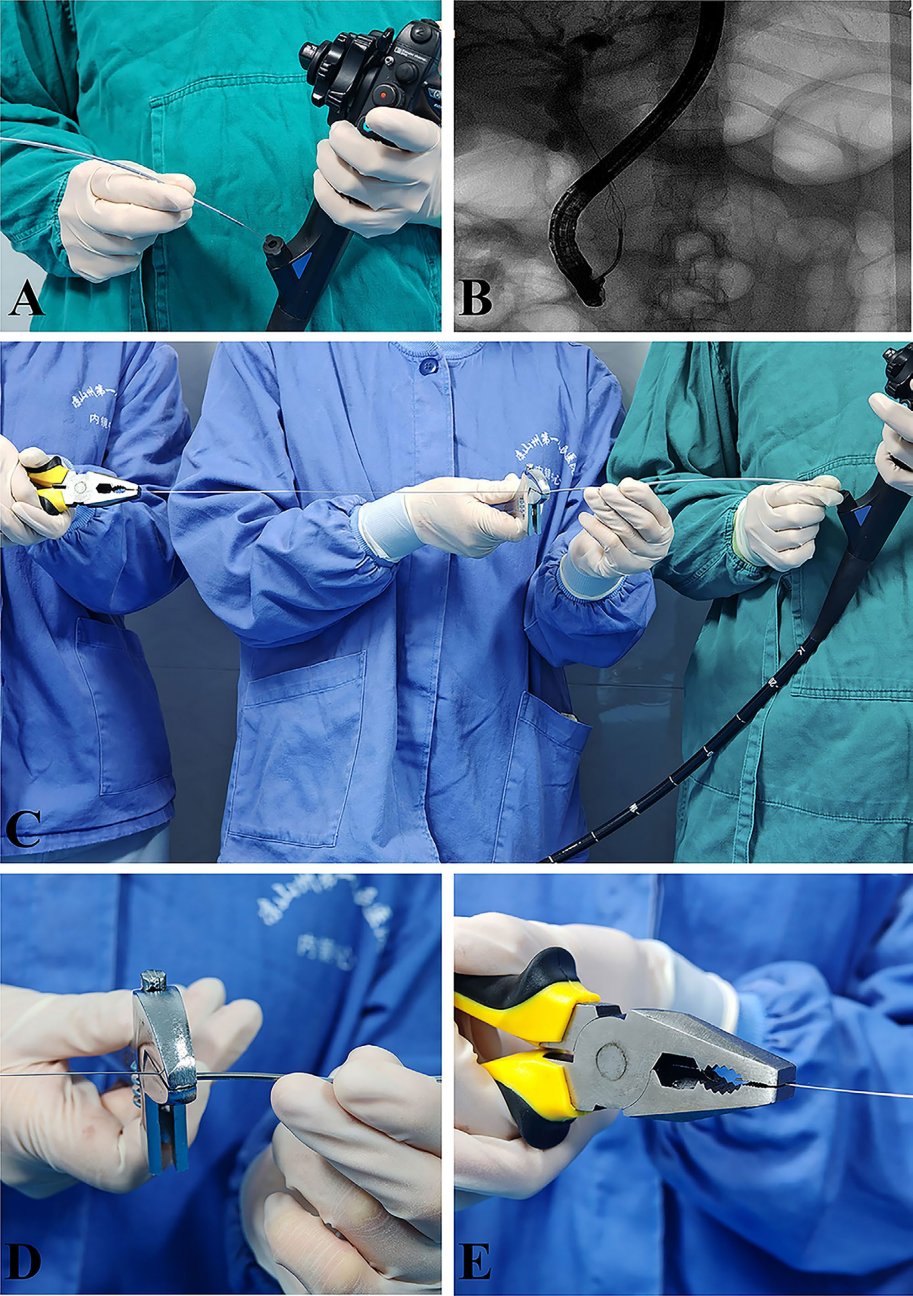
(A) Inserting the metal outer sheath of the lithotripter along the inner core of the retrieval basket through the accessory channel of the duodenoscope into the duodenal papilla and reaching the common bile duct. (B) The outer sheath of the lithotripter arrived at the location of the stone. (C) With the assistance of an assistant, tighten the inner core of the retrieval basket for mechanical lithotripsy. (D) Using a thumb forceps to tighten the inner core of the retrieval basket. (E) Using a wrench to secure the metal outer sheath to prevent its outward movement.

Although basket impact is a rare complication of ERCP, it is still unavoidable. A salvage device is usually the preferred option. However, due to the extremely low incidence of basket impaction, many medical institutions may not have salvage devices. On the contrary, most medical institutions have integrated lithotripters, which could be modified as effective salvage devices.

Most salvage devices cannot pass through the instrument channel of the duodenoscope; it is easy to cause papilla damage, resulting in the injury of the duodenal papilla and affecting further endoscopic operation or inducing postoperative pancreatitis, as well as bleeding, perforation, and other complications. However, our modified technique with the modified device allows safe passage through the papilla into the CBD via the instrument channel of the duodenoscope under direct endoscopic view.

Reviewing the previous literature, past cases often faced challenges with traction wires fractured near the handle; then the basket wire could not connect to the handle and could not continue to try the second lithotripsy. When earlier attempts using a second lithotripter to entrap the impacted first basket failed, exchanging the initial metal sheath for shorter ones allowed the immediate continuation of lithotripsy in most cases and offered more opportunities using the same fractured wire [1]. However, specific metal sheathes are required. In our approach, we used the shortened metal sheath and tried to crush stones multiple times using the same fractured wire, offering an immediate, easy, timesaving, cost‐effective solution.

## Author Contributions

**Peixi Liu:** methodology. **Qin Zhang:** supervision. **Yufan Ma:** writing – original draft.

## Ethics Statement

The studies involving human participants were reviewed and approved by The first people's Hospital of Liangshan Yi Autonomous Prefecture Academic Ethics Committee.

## Consent

The authors provided the written informed consent to participate in this study.

## Conflicts of Interest

The authors declare no conflicts of interest.

## Data Availability

The data that support the findings of this study are available on request from the corresponding author. The data are not publicly available due to privacy or ethical restrictions.
